# Cooperative Oxygen Sensing by the Kidney and Carotid Body in Blood Pressure Control

**DOI:** 10.3389/fphys.2017.00752

**Published:** 2017-10-04

**Authors:** Daniela Patinha, Wioletta Pijacka, Julian F. R. Paton, Maarten P. Koeners

**Affiliations:** ^1^School of Physiology, Pharmacology and Neuroscience, Biomedical Sciences, University of Bristol, Bristol, United Kingdom; ^2^Institute of Biomedical and Clinical Science, University of Exeter Medical School, University of Exeter, Exeter, United Kingdom

**Keywords:** hypoxia, kidney, carotid body, hypertension, angiotensin II

## Abstract

Oxygen sensing mechanisms are vital for homeostasis and survival. When oxygen levels are too low (hypoxia), blood flow has to be increased, metabolism reduced, or a combination of both, to counteract tissue damage. These adjustments are regulated by local, humoral, or neural reflex mechanisms. The kidney and the carotid body are both directly sensitive to falls in the partial pressure of oxygen and trigger reflex adjustments and thus act as oxygen sensors. We hypothesize a cooperative oxygen sensing function by both the kidney and carotid body to ensure maintenance of whole body blood flow and tissue oxygen homeostasis. Under pathological conditions of severe or prolonged tissue hypoxia, these sensors may become continuously excessively activated and increase perfusion pressure chronically. Consequently, persistence of their activity could become a driver for the development of hypertension and cardiovascular disease. Hypoxia-mediated renal and carotid body afferent signaling triggers unrestrained activation of the renin angiotensin-aldosterone system (RAAS). Renal and carotid body mediated responses in arterial pressure appear to be synergistic as interruption of either afferent source has a summative effect of reducing blood pressure in renovascular hypertension. We discuss that this cooperative oxygen sensing system can activate/sensitize their own afferent transduction mechanisms via interactions between the RAAS, hypoxia inducible factor and erythropoiesis pathways. This joint mechanism supports our view point that the development of cardiovascular disease involves afferent nerve activation.

## Introduction

Oxygen is essential for aerobic metabolism, a fundamental mechanism for energy production. However, the delivery of optimal levels of oxygen to tissues must be highly regulated as both insufficient (hypoxia) or excessive oxygen levels (hyperoxia) are highly detrimental. Indeed, tissue oxygenation has been found to be reduced during pathological conditions such as cancer (Liu et al., 2016), diabetes (Palm et al., 2003), hypertension (Welch et al., 2001), chronic kidney disease (Milani et al., 2016), and stroke (Ferdinand and Roffe, 2016). We will explore the idea that an inappropriate activation of some of the signaling pathways that counteract hypoxia can contribute to the development of hypertension and cardiovascular disease through activation of the sympathetic nervous system.

Adaptation to low partial pressure of oxygen, for instance at high altitude, triggers a protective mechanism that includes an increase in sympathetic activity, vascular resistance, and blood pressure (Hainsworth and Drinkhill, 2007). The kidney and carotid body both participate in this adaptation for the maintenance of systemic oxygen levels and blood flow (Marshall, 1994; Dunn et al., 2007; Jelkmann, 2011). For example, within the kidney the number of erythropoietin-producing cells increases proportionally to the degree of hypoxia, which correlates directly with the concentration of erythropoietin in blood (Koury and Haase, 2015), ensuring higher blood oxygen carrying capacity (Marshall, 1994; Dunn et al., 2007; Jelkmann, 2011). In comparison, the carotid body triggers reflex increases ventilation and sympathetic activity to maintain oxygen tension and delivery (Marshall, 1994). Interestingly the kidney and the carotid body are both innervated by efferent and afferent nerve fibers and are both targets and modulators of sympathetic activity. Hypoxic-hypoperfusion of the kidney and carotid body is a likely trigger for increased reflex sympathetic activity (Koeners et al., 2016) and aberrant afferent drive from these organs is implicated in the etiology of neurogenic hypertension (Fisher and Paton, 2012; Narkiewicz et al., 2016; Silva et al., 2016; Osborn and Foss, 2017).

We wish to explore whether there is cooperative oxygen sensing between the kidney and the carotid body that plays a role in homeostasis. We will consider this notion under physiological conditions where it counteracts moderate or brief tissue hypoxia over an acute short time scale (minutes to hours). We will also assess the long term (days) cooperative oxygen sensing where hypoxia afferent signaling from these organs persists and drives the progression of cardiovascular disease. We initiate our discussion by examining the effect of short and long term hypoxia on the kidney and carotid body.

## Hypoxia, afferent nerve activation/sensitization and hypertension

### Hypoxia

Oxygen delivery to different organs is a product of cardiac output and arterial oxygen content per unit of time (Habler and Messmer, 1997; Leach and Treacher, 2002). The majority of oxygen transported throughout the body is reversibly bound to hemoglobin, and its diffusion to the cell is dependent on the local tissue partial pressure gradient. Oxygen consumption in a given tissue is the volume of oxygen consumed per unit of time, which in aerobic conditions corresponds to the metabolic rate [adenosine triphosphate (ATP) formation/consumption] (Habler and Messmer, 1997; Leach and Treacher, 2002). Each organ has a different metabolic rate, and hence a different oxygen demand. Every organ has the capacity of altering their metabolic rate (except skin), as part of the local dynamics which, in most cases, directly influences local blood flow. Due to an oxygen reserve, oxygen consumption is independent of oxygen delivery within a wide range of delivered oxygen. In addition, organs have different oxygen extraction ratios (fraction of oxygen delivery) and different oxygen reserves. Organs that have a lower oxygen extraction, such as the skin, have a higher oxygen venous reserve. Conversely, the heart and the brain have a limited oxygen reserve due to the high oxygen extraction (Habler and Messmer, 1997). In case of systemic low oxygen delivery or systemic high oxygen demand, blood can be redistributed to sustain high extraction organs, without compromising oxygen supply to the ones with higher oxygen reserves. However, increases in oxygen consumption or decreases in oxygen delivery will increase oxygen extraction to maintain aerobic metabolism. When the critical oxygen delivery limit is reached, any increase in oxygen consumption or decrease in oxygen delivery will lead to tissue hypoxia, as reviewed in Leach and Treacher (2002).

The term hypoxia represents a reduced partial pressure of oxygen and deoxygenation of tissue. Such a condition triggers a series of responses that manifest themselves over different sequential time frames: First, acute systemic reduction of tissue oxygen partial pressure stimulates peripheral chemoreceptors that triggers respiratory and cardiovascular responses to elevate oxygen uptake and delivery to bodily organs (Lahiri et al., 1980; Marshall, 1994; Blessing et al., 1999). Second, persistent subacute hypoxia activates cellular pathways through the stabilization of hypoxia inducible factor (HIF)-1 and HIF-2 complexes (Greer et al., 2012). HIF-1 is understood to be the most important regulator of cellular responses to hypoxia. This long term adaptation is triggered in order to enhance the oxygen delivery capacity and maintain organ function, including glycolysis, angiogenesis, erythropoiesis, iron metabolism, pH regulation, apoptosis, cell proliferation as well as cell-cell, and cell-matrix interactions (Haase, 2006; Greer et al., 2012). Examples of classic HIF target genes are phosphoglycerate kinase-1, glucose transporter-1, vascular endothelial growth factor, and erythropoietin. Pathologic conditions like renal disease and diabetic nephropathy have shown to impede this adaptation via, for example, desensitization of renal erythropoietin-producing cells by uremic toxins (Chiang et al., 2012) or direct inhibition of HIF activity (Nordquist et al., 2015; Tanaka et al., 2016). Indeed, treatment which increased HIF activity corrected abnormal renal metabolism (oxygen consumption, efficiency) and hemodynamics (renal blood flow, glomerular filtration) in a rat model of chronic kidney disease (Deng et al., 2010). Interestingly these effects were similar to RAAS inhibition but involved a significantly different molecular pathway. Third, chronic sustained tissue hypoxia can result from stenosis/partial occlusion of conduit arteries that may be of congenital or atherosclerotic origin. In case of obstruction of blood flow ischemic injury will follow due to the reduced nutrient and oxygen supply. As proposed previously (Koeners et al., 2016), hypoxic-hypoperfusion may trigger aberrant renal and/or carotid body afferent tonicity and initiate/amplify sympathetic hyperactivity accentuating arteriolar vasoconstriction and further compounding blood flow and oxygen delivery; this results in hypertension.

### Renal oxygenation

Renal oxygenation is tightly regulated (both short and long term) to maintain the balance between oxygen supply and demand. Under normal conditions, but under anesthesia, renal partial pressure of oxygen varies from 15 to 50 mmHg in the cortex and 5–25 mmHg in the renal medulla (Evans et al., 2008; Carreau et al., 2011). Due to the unique anatomy of the kidney, the renal medulla is believed to receive the minimum level of oxygen needed to support normal cell function, and hence might be very susceptible to reductions of the partial pressure of oxygen.

The heterogeneous oxygenation within the renal parenchyma is a result of the different tasks performed along the nephron and is mainly associated with: (1) the high energy demand necessary to reabsorb Na^+^, (2) the arteriovenous oxygen shunt, and (3) the requirements to perform the countercurrent mechanism that permits urine concentration. Almost all of the renal oxygen consumption is coupled with active Na^+^ transport through Na-K-ATPase (Mandel and Balaban, 1981). To put this in perspective, the energy required to reabsorb 1 mol Na^+^ is ~7 kj, which corresponds to lifting 1 mol Na^+^ (~20 g) to a height of 70 km (Hansell et al., 2013). In addition, the development of a Na^+^ gradient allows the transport of other molecules such as glucose, amino-acids, other solutes, and water. Since the reabsorption of Na^+^ depends on the glomerular filtration rate, increasing the blood flow to the kidney will increase the filtered Na^+^ load and further deplete renal oxygen due to a higher oxygen consumption (Hansell et al., 2013). Another factor contributing to the oxygen content in the renal tissue is the arteriovenous oxygen shunt. The renal arteries and veins run in close proximity, oxygen can diffuse in such a way that the oxygen content in the veins is higher than that in the glomerular capillaries and efferent arterioles (Schurek et al., 1990; Welch et al., 2001; Evans et al., 2008). This is especially important when considering that the renal medullary peritubular capillaries arise from the efferent arterioles of the juxtamedullary glomeruli. This vessel network also has a low blood flow to maintain the gradients necessary for the countercurrent mechanism that allows urine concentration (Brezis and Rosen, 1995; Fry et al., 2014). Furthermore, the close proximity of the ascending and descending medullary vasa recta may theoretically promote more arteriovenous oxygen diffusion (Zhang and Edwards, 2002). Hence, the renal medulla has a relative low partial pressure of oxygen and is highly susceptible to ischemic/hypoxic injury.

As already indicated above the kidney contributes to long-term (days) hypoxic adaptation. It has a primordial role in maintaining systemic oxygen content through hypoxia-induced erythropoietin production from the renal interstitial fibroblast-like cells. Under hypoxia, HIF-α is no longer hydroxylated, and HIF-α subunits can accumulate to activate HIF-1-dependent genes like erythropoietin and many others (Haase, 2006). Erythropoietin acts on bone marrow to increase red blood cell production (Dunn et al., 2007; Jelkmann, 2011) which will increase the oxygen carrying capacity. Therefore, the kidney serves as one of the most important physiological oxygen sensors and detectors of systemic hypoxia.

### Renal hypoxia, afferent nerve activation/sensitization, and hypertension

Chronic hypoxia has been confirmed in different kidney disease models such as diabetic nephropathy (Palm et al., 2003) and hypertension (Welch et al., 2001). Long term renal hypoxia is an increasingly recognized common pathway for the development of chronic kidney disease (Hansell et al., 2013; Kawakami et al., 2014), but it can also generate renal injury. Friederich-Persson et al showed that increasing kidney oxygen metabolism, using a mitochondrial uncoupler, reduces the cortical partial pressure of oxygen and causes proteinuria in otherwise healthy rats (Friederich-Persson et al., 2013).

Acute renal hypoxia may also be involved in the activation of renal afferent pathways that leads to the establishment and maintenance of elevated blood pressure. The cell bodies of the renal afferent nerve fibers are located in the dorsal root ganglia and project to the ipsilateral dorsal horn where they synapse with neurons projecting to sites associated with cardiovascular regulation such as the nucleus tractus solitarii and the rostral ventral medulla (Solano-Flores et al., 1997; Ciriello and de Oliveira, 2002; Kopp, 2015) where integration with other inputs will occur and reflex sympathetic responses can be generated. Indeed, perfusion of the kidney with hypoxic blood (PaO_2_: 36 mmHg) is enough to increase femoral perfusion pressure by >30 mmHg. This response is mediated by renal afferent nerves as it was abolished after denervating the kidney (Ashton et al., 1994). However, whether there is a threshold, or graded thresholds of renal tissue partial oxygen pressure for renal afferent nerve activation is unknown. Furthermore, performing the same experiment using normoxic blood and ischemic metabolites such as bradykinin, prostaglandin E2, and adenosine elicits similar rises in blood pressure (Ashton et al., 1994). This demonstrates that both low partial pressure of oxygen and ischemic metabolites can directly and/or indirectly stimulate renal sensory nerve fibers, promoting reflex increase of the sympathetic nerve activity, and blood pressure (Katholi et al., 1985).

In the two-kidney one clip model of hypertension, denervation of the hypoperfused (clipped) kidney reduced arterial blood pressure, noradrenaline plasma concentration and peripheral sympathetic nerve activity (Katholi et al., 1982). Similarly, in the one-kidney, one-clip model of renovascular hypertension, dorsal root rhizotomy ipsilateral to the clipped kidney attenuated the evoked hypertension (Wyss et al., 1986). Importantly, even a small lesion in the kidney that results in an area(s) of ischemia (hypoperfusion) not necessarily affecting renal function, e.g., by intrarenal injection of phenol, can cause neurogenic hypertension via activation of hypoxia-sensitive renal afferent mechanisms (Ye et al., 2002; Koeners et al., 2014). In this phenol model of renal neurogenic hypertension there is a rapid (within 5 min) and sustained increase in blood pressure that is abolished by nephrectomy or denervation of the injured kidney (Ye et al., 2002; Koeners et al., 2014). These studies support the concept that hypoxia-induced renal afferent activation contributes to hypertension by increasing sympathetic nerve activity through reflex pathways. Similarly, in patients with renovascular hypertension, restoration of renal perfusion reduces muscle sympathetic nerve activity and blood pressure (Miyajima et al., 1991) and renal nerve ablation can reduce blood pressure and muscle sympathetic nerve activity in some patients with resistant hypertension (Hering et al., 2014). Finally, given the change in set-point of sympathetic activity and blood pressure it is perhaps not surprising that the baroreceptor reflex is reset and gain improved following renal denervation in a rat model of chronic kidney disease (Chen et al., 2016).

We suggest that the aforementioned renal afferent reflex pathway impinging on the nucleus tractus solitarii is a likely nodal point for modulation of the baroreflex. Taken together, both acute or chronic renal hypoxia and hypoperfusion (associated with macro- or microvascular disease) may cause/sustain hypertension through activation of renal afferent chemosensory fibers (Campese et al., 2006; Johns et al., 2011; Foss et al., 2015; Banek et al., 2016). This has parallels with sustained activation of the peripheral chemoreceptors, which are considered next.

### Carotid body oxygenation

Carotid bodies are distinct organs located bilaterally at the bifurcation of the common carotid arteries. They have the highest blood flow per tissue weight when compared to any other organ in the body and play an important role in the monitoring and maintenance of physiological levels of blood gases through reflex activation of respiration (Lahiri et al., 1980). The carotid body consists of glomus or type I cells, which are the primary oxygen sensing cells, and supporting or type II cells. Blood supply to the carotid body originates mostly from the carotid artery. The carotid body vasculature is innervated by postganglionic sympathetic fibers from the superior cervical ganglion and by parasympathetic fibers originating from intraglomic ganglion cells. With larger blood vessels having predominantly parasympathetic innervation and smaller blood vessels having predominantly sympathetic innervation, as reviewed by Kumar and Prabhakar (2012). Hence, the arterioles that are in close contact with the type I and type II cells are predominantly innervated with sympathetic fibers, thus more prone to vasoconstriction/hypoperfusion that promotes chemoreceptor activation.

The blood supply to the carotid body is very high given the total metabolic demand, with <3% of oxygen consumed (De Burgh Daly et al., 1954). Of interest, tissue partial pressure of oxygen is lower than that measured in the venous blood, suggesting the existence of an arteriovenous shunt, with potentially a large amount of blood bypassing the chemosensory cells (Acker et al., 1971; Acker and O'Regan, 1981; O'Regan et al., 1990). Despite the very low total organ oxygen consumption, type I cells have a very high metabolic rate with an oxygen consumption at rest approaching the maximum (Duchen and Biscoe, 1992). This very high oxygen consumption makes type I cells very sensitive to reductions in partial pressure of oxygen.

The microvascular partial pressure of oxygen in the carotid body is around 50–70 mmHg in the anesthetized cat (Whalen et al., 1973; Rumsey et al., 1991). It has been shown that changes in oxygenation below this level results in a powerful increase in carotid body afferent activity (Vidruk et al., 2001). Neurosecretion from the glomus cells within the carotid body in response to acute hypoxia is fundamental to chemosensation and involves release of a variety of molecules including acetylcholine, dopamine, ATP, and neuropeptides such as substance P or enkephalins have been investigated. Recently, evidence for gas signaling molecules such as nitric oxide and carbon monoxide have been highlighted in the carotid body for oxygen sensing (Prabhakar, 2000; Nurse and Piskuric, 2013). These transmitters all activate the terminals of afferent fibers at the glomus cell-afferent junction. Anatomical studies on the cat carotid region revealed that glomus cells are innervated both by sensory and autonomic fibers mostly from the carotid sinus nerve but also by superior cervical ganglion and occasionally the ganglio-glomerular nerves (Eyzaguirre and Uchizono, 1961; Knoche and Kienecker, 1977).

### Carotid body hypoxia, afferent nerve activation/sensitization, and hypertension

Chemoreceptor activation typically occurs after a change in arterial partial oxygen pressure from ~95 to ~50 mmHg for a single unit chemoreceptor *in vitro* (Vidruk and Dempsey, 1980), and to ~35 mmHg for a whole nerve *in vivo* (Vidruk et al., 2001). However, chemoreceptor afferent fibers show huge variability in their threshold of activation to hypoxia permitting graded responses (Vidruk et al., 2001) and therefore is likely to overlap with renal afferent threshold(s). It has also been proposed that carotid body glomus cells and associated sensory fibers have reflex specific circuits that account for different patterns of response evoked by different stimulants or different levels of hypoxia (acute or chronic) (Paton et al., 2013). Importantly, the afferent nerves of the different sub-populations of glomus cells may project into compartmentalized sites of the nucleus tractus solitarii that regulate cardiac, respiratory, sympathetic as well has higher brain functions (Paton et al., 2013). Carotid body chemoreceptor activation leads to an increased sympathetic tone through glutamatergic excitatory signaling in the nucleus tractus solitarii, rostral ventrolateral medulla, and the paraventricular nucleus resulting in increased blood pressure (Marshall, 1994; Blessing et al., 1999).

The carotid chemoreflex plays a powerful role in the blood pressure regulation including modulation of renal function. For example, carotid chemoreflex activation using autologous venous blood, while maintaining carotid sinus pressure constant, reduced renal blood flow, and glomerular filtration rate through increased renal nerve activation in dogs (Karim et al., 1987). For a long time it has been considered that carotid bodies only change blood pressure over seconds. However, recently an increasing amount of evidence suggests that persistent stimulation of the carotid body might play a role in long-term blood pressure control. In hypertensive animals and humans, chemo-sensory fibers are continuously activated causing increased vasomotor sympathetic activity and hypertension in animals and humans (Sinski et al., 2014; Pijacka et al., 2016b). To demonstrate carotid body tonicity, the carotid sinus nerves were resected and this was found to attenuate the developmental increase in the blood pressure in young spontaneously hypertensive animals (Abdala et al., 2012). In addition, carotid sinus denervation performed in adult spontaneously hypertensive rats reduced blood pressure and sympathetic activity chronically; it also led to increased aortic baroreflex sensitivity (Abdala et al., 2012; McBryde et al., 2013). These animal studies were translated into a first human study with similar results in some hypertensive patients (Narkiewicz et al., 2016).

This evidence suggests that excessive afferent signaling from carotid bodies may lead to the development of pathological conditions such as hypertension in animals and human. However, what triggers carotid body tonicity is still poorly understood but it is unlikely to be *systemic* hypoxia. The possibility that the carotid body is chronically hypoxic, perhaps due to hypoperfusion secondary to either increased sympathetic vasomotor tone or circulating angiotensin II is plausible, at least in hypertension.

## Kidney and carotid body: cooperative oxygen sensors

As outlined above acute and chronic hypoxia is sensed by both the kidney and the carotid body that activates afferent nerve signaling promoting reflex increases in sympathetic nerve activity triggering hypertension (Katholi et al., 1982; Tafil-Klawe et al., 1985; Somers et al., 1988; Ashton et al., 1994; Ye et al., 2002; Campese et al., 2006; Tan et al., 2010; Johns et al., 2011; Abdala et al., 2012; Sinski et al., 2012; McBryde et al., 2013; Paton et al., 2013; Koeners et al., 2014; Foss et al., 2015; Banek et al., 2016; Pijacka et al., 2016a,b). We hypothesize that the response to systemic hypoxia is based on both local renal and carotid body specific chronic hypoxia sensing which act cooperatively (see Box 1). Given the greater sensitivity of the kidney to hypoxia (see above) we propose that this organ responds first to falls in arterial oxygen tension. As oxygen tension falls further signals cascading from the kidney activate the carotid body that once recruited acts cooperatively to ensure sustained long term sympathoexcitation. Evidence for this cooperative mechanism comes from the additive blood pressure lowering effect after renal denervation is performed in combination with carotid body de-afferentation/resection (McBryde et al., 2013, 2017; Pijacka et al., 2016a). The carotid sinus and the renal afferent nerves converge in multiple central cardiovascular regulation areas, providing an anatomical basis for interaction such as the nucleus tractus solitarii and the rostral ventrolateral medulla (Johns et al., 2011).

Box 1 **Novel insights of the cooperative oxygen sensing by the kidney and carotid body in blood pressure control.**

- Integration of renal and carotid body afferent activity act together to regulate blood pressure during both acute and chronic hypoxia.
- The interaction between the kidney and the carotid body is cooperative—not facilitatory or occlusive.
- The afferent systems of the kidney and the carotid body may have overlapping thresholds for detecting reduced tissue oxygen partial pressure.
- Given the postulated overlap in thresholds, there may be a temporal sequence to the reflex responses elicited between the two organs.
- The cooperative oxygen sensing by the kidney and carotid body could be of great relevance in the pursuit of novel ways to treat diseases in which there is sympathetic overdrive.

### Lines of communication: kidney to carotid body

**Figure 1:**
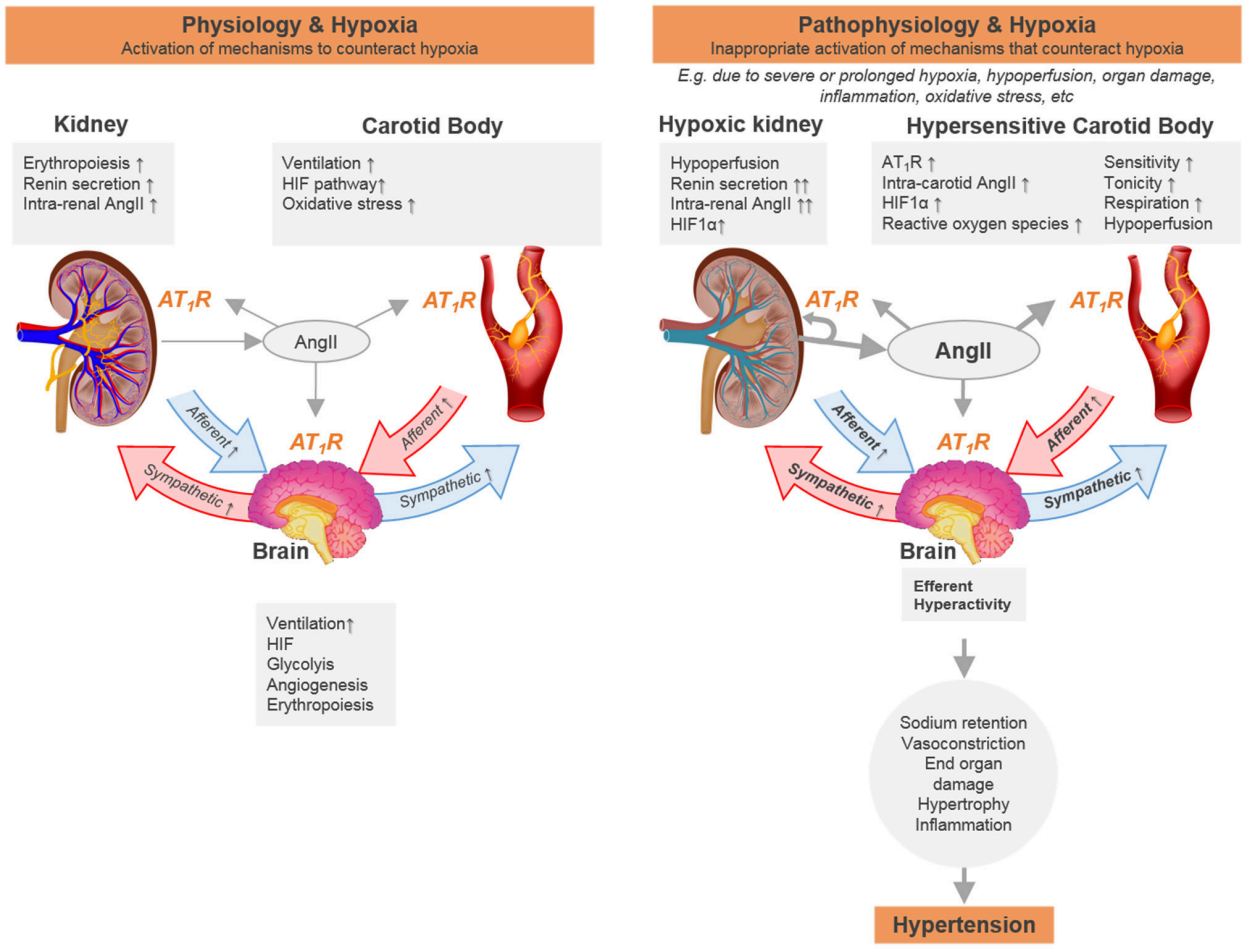
Schematic representation of the hypothesis that chronic hypoxia sensed by carotid body and kidney is essential for physiological adaptation and when over-activated can contribute to cardiovascular disease due to positive cross-organ interactive feedback mechanisms. We propose a temporal sequence to the reflex responses elicited between the two organs. One potential way of how the transition from one state to the other would occur includes the inability of the kidney to overcome tissue hypoxia during pathological conditions related to hypoperfusion and/or increased metabolic rate (e.g., vasoconstriction, mitochondrial dysfunction, hyperfiltration). Signals cascading from the hypoxic kidney activate the carotid body that acts cooperatively to ensure sustained (and in the end aberrant) long term sympathoexcitation. Furthermore, the renin angiotensin system is activated in both organs in response to low blood flow/hypoxia. This chronic low blood flow/hypoxia together with the activation of the renin angiotensin system forms a non-functional positive feedback loop that leads to tissue damage. Increasing the renin angiotensin system will lead to activation of different pathways to ensure proper oxygen delivery, including hypoxia inducible factor and erythropoiesis, that may also contribute to the dysfunctional sympathetic activation in hypertension.

Given their relative sensitivities to acute and chronic hypoxia it would seem logical to postulate a communication cascade from the kidney to the carotid body (Figure 1). This might include the RAAS. The RAAS plays a key role in cardiovascular and renal physiology and is primarily activated as a functional response to maintain organ perfusion. Most of the RAAS effects arise from angiotensin II AT_1_ receptor activation and include direct vasoconstriction, increased tubular sodium reabsorption, activation of sympathetic nervous system and increased aldosterone release, fibrosis, reactive oxygen species production and cell proliferation (Balakumar and Jagadeesh, 2014). Accordingly, the RAAS is currently the main pharmacological target of anti-hypertensive therapy (Romero et al., 2015). The mechanism of action of RAAS blockade seems to be straightforward: reduce or block angiotensin II and aldosterone, thereby preventing the deleterious cardiovascular effects. Strikingly, RAAS inhibition is also effective in patients with medium-to low plasma RAAS activity (Te Riet et al., 2015). Moreover, in some cases, after inhibiting angiotensin II/aldosterone receptors, plasma levels of these two hormones returns to normal or even rise above pre-treatment levels: the so-called angiotensin II escape/ refractory hyperaldosteronism (Te Riet et al., 2015). Nonetheless, RAAS inhibition remains partially anti-hypertensive (Te Riet et al., 2015), which may be related to locally generated and regulated RAAS. Experimental evidence shows that intra-renal RAAS is compartmentalized from systemic RAAS; for example, intrarenal RAAS is not adequately inhibited by plasma concentrations of RAAS inhibition in currently used dosages (Nishiyama et al., 2002). Whether this RAAS compartmentalization occurs in other organs, like the carotid body, and is immune to systemic RAAS antagonists is unknown.

If the carotid body is an additional source of afferent drive contributing to sympathetic excess in conditions of hypertension, then what drives it? Certainly, all the components of the RAAS have been identified in the carotid body, except renin (Allen, 1998; Lam and Leung, 2002, 2003). Interestingly a high density of angiotensin II AT1 receptors are located on the primary chemoreceptor element, the glomus cell (Allen, 1998) and their expression and function is upregulated when exposed to chronic hypoxia (Leung et al., 2000). We hypothesize that this forms a line of communication to amplify the generation of sympathetic activity. We do not rule out that in renovascular hypertension heightened sympathetic activity to the carotid body itself (causing vasoconstriction, hypoperfusion) results in enhanced carotid body discharge and elevated systemic RAAS activity. We will address the proposed role of RAAS and cooperative oxygen sensing in the kidney and carotid body.

#### Hypoxia and renal renin-angiotensin aldosterone system in hypertension

Renal sympathetic activation constricts the renal vasculature thus reducing renal blood flow and glomerular filtration rate, increases sodium retention, and activates the RAAS through increased renin release from the juxtaglomerular cells (DiBona, 2000; Johns et al., 2011). Angiotensin II AT_1_ receptor activation affects oxygen availability in the kidney by acting on both its delivery (vasoconstriction) and consumption (increased metabolic rate, decreased efficiency, or both). Angiotensin II-induced reduction in renal blood flow is associated with the reduction of partial pressure of oxygen in the renal cortex (Welch et al., 2005; Emans et al., 2016). Interestingly, in the two kidney one clip model of renovascular hypertension, renal angiotensin II is increased in both kidneys from the first week post clipping (Sadjadi et al., 2002). This suggests that the induced hypoxic-hypoperfusion in the ipsi-lateral kidney also activates the RAAS in the contra-lateral kidney (perhaps via a renorenal reflex) in the development of renovascular hypertension in this model.

By increasing angiotensin II within the kidney, HIF, and erythropoiesis pathways may be triggered to increase oxygen delivery systemically. This in line with the fact that angiotensin II infusion reduces cortical partial pressure of oxygen (Welch et al., 2005; Emans et al., 2016) and increases erythropoietin production in the kidney (Gossmann et al., 2001; Jelkmann, 2011; Calo et al., 2015). Once active, the HIF and erythropoiesis pathways act as feedforward mechanisms. For instance, the increased renal angiotensin II further exaggerates the efferent sympathetic input and sodium retention by abolishing the renorenal reflex, as reviewed by Johns et al. (2011). We cannot exclude that aldosterone also plays a role in our hypothesized cooperative oxygen sensing and blood pressure control. Increased aldosterone secretion is associated with hypertension (Laragh et al., 1960; Mackenzie and Connell, 2006). Clinical studies have shown that aldosterone blockade is the most effective add-on drug (step 4 treatment in the NICE guidelines; www.nice.org.uk/Guidance/CG127) for the treatment of resistant hypertension (Epstein and Duprez, 2016). However, the role of the mineralcorticoid receptor in relation with cooperative sensing of hypoxia by the kidney and carotid body is completely unknown.

Taken together, activation of renal afferents (discussed previously) and the RAAS act as two distinct feedback systems during acute and chronic hypoxia sensing by the kidney. As hypoxia is relative to physiological tissue oxygen pressure these feedback systems are likely to have overlapping hypoxia thresholds for their activation and play intricate roles in both acute and/or chronic changes in tissue oxygenation.

#### Carotid body and renin-angiotensin aldosterone system in hypertension

Locally generated angiotensin II increases carotid body afferent discharge (Lam and Leung, 2002) and increases the intracellular calcium levels via activation of AT_1_ receptors in carotid body type I (Fung et al., 2001) and type II (Murali et al., 2014) cells. Murali and coworkers hypothesized that angiotensin II AT_1_ receptor-mediated pannexin-1 channel dependent ATP release in type II cells serves as a boost for carotid body excitation (Murali et al., 2014), which may be specially relevant in conditions where local angiotensin II is elevated such as chronic heart failure (Li et al., 2006), sleep apnea (Lam et al., 2014), and in the hypertensive state. Chronic hypoxia induces angiotensin II AT_1_ receptor expression in the carotid body (Lam et al., 2014). Blockade of angiotensin II AT_1_ receptors prevents chronic intermittent hypoxia-mediated reactive oxygen species production in the carotid body (Lam et al., 2014) and the development of hypertension (Fletcher et al., 1999). In fact, angiotensin II AT_1_ receptor activation has been shown to induce sensory long term facilitation of the carotid body via NADPHoxidase (Peng et al., 2011b). Importantly, in chronic intermittent hypoxia, carotid body afferent nerve activation is also mediated by angiotensin II AT_1_ receptors (Marcus et al., 2010). Moreover, reducing the blood flow (hypoxic-hypoperfusion) to carotid body by carotid artery occlusion elevated angiotensin II AT_1_ receptor expression in carotid body and increased chemoreceptor activity in the rabbit (Ding et al., 2011). Activation of angiotensin II AT_1_ receptor *in vitro* (hence independently of vasoconstriction) by AngII activated afferent chemoreceptor activity (Allen, 1998). Importantly, blocking angiotensin II AT_1_ receptor receptors in isolated carotid body blunts angiotensin II AT_1_ receptor -dependent carotid body sensitivity (Li et al., 2007).

Many mechanisms govern carotid body signaling, including ATP-gated ion channels (called purinergic P2X receptors), specifically the C-fiber-localized, P2X3-receptor subtypes, which are commonly associated with afferent sensitization and might contribute to hyper-reflexic disease states in a variety of organs. We found that in spontaneously hypertensive rats P2X3 receptors are upregulated and that blockade of P2X3 receptors was effective at reducing blood pressure and sympathetic activity in the spontaneously hypertensive rats but had no effect in normotensive control rats (Pijacka et al., 2016b). Interestingly chronic angiotensin II infused hypertensive rats have upregulated intrarenal P2X1 receptors (Franco et al., 2011).

Taken together this underlines the important role RAAS in carotid body can play in hypoxia sensing, possibly via purineric signaling. Potentially the kidney could trigger the carotid body via RAAS activation, compounding renal sympathetic activity (driving renal afferents) which will exaggerate RAAS activity. If this is true a continued carotid body drive could be deleterious to the kidney causing over excitation of renal afferents, genomic changes, resulting in a double wind up of the systems and ultimately cause persistent hypertension.

## Commonality in the HIF pathway and its role in cooperative oxygen sensing by the kidney and carotid body

When tissue oxygen levels drop chronically, expression of the HIF-1α and−1β subunits increase. The HIF-1 α/β heterodimer binds and activates expression of various genes including those encoding glycolytic enzymes (for anaerobic metabolism), vascular endothelial growth factor (for angiogenesis), inducible nitric oxide synthase and heme oxygenase-1 (for production of vasodilators), erythropoietin (for erythropoiesis), and possibly tyrosine hydroxylase (for dopamine production to increase breathing) (Guillemin and Krasnow, 1997). These genes help the cell survive at low oxygen and act to restore normal oxygen levels.

In the normal, fully developed kidney, HIF-1α is expressed in most cell types, whereas HIF-2α is mainly found in renal interstitial fibroblast-like cells and endothelial cells. The HIF pathway has been implicated with renal development, normal kidney function, and disease (Haase, 2006). Recently HIF-1α mRNA has been suggested to be a potential biomarker in chronic kidney disease, and comes primarily from cells of renal origin (Movafagh et al., 2017). Interestingly, the carotid body glomus cells constitutively overexpress HIFs and certain HIF transcriptional targets that are normally part of the counteractive mechanism against the negative impacts of sustained hypoxia (Zhou et al., 2016). Specifically, the glomus cells transcriptionally upregulate atypical mitochondrial electron transfer chain components, suggesting unique mitochondria are present in the carotid body and may be responsible for oxygen sensing (Zhou et al., 2016).

A few years ago, Gassmann and Soliz postulated that there was a crosstalk between the ventilatory and erythropoietin responses and suggested that the chemoreflex pathway may be activated by circulating erythropoietin (Brines et al., 2004; Gassmann and Soliz, 2009). In fact, circulating erythropoietin, acting on its receptors present in the carotid body improves the hypoxic ventilatory response (Soliz et al., 2005) suggesting a key role of erythropoietin for hypoxia adaption beyond the classical regulation of erythropoiesis (Pichon et al., 2016). Interestingly, in models of chronic and intermittent hypoxia, erythropoietin and its receptor are upregulated in the carotid body which may promote enhanced excitability and contribute to the pathophysiology of breathing disorders (Lam et al., 2009).

Balanced expression of the HIF-α isoforms is essential for the correct functioning of oxygen sensing in the carotid body (Yuan et al., 2013; Prabhakar and Semenza, 2016). HIF-1α is expressed in both type I and type II cells of the carotid body, while HIF-2α is only expressed in type I cells (Roux et al., 2005). The carotid body chemoreflex response to acute and chronic hypoxia is blunted when HIF-1α expression is reduced (Kline et al., 2002; Yuan et al., 2011, 2013). Conversely, acute and chronic hypoxic sensitivity is enhanced when HIF-2α is reduced (Nanduri et al., 2009; Peng et al., 2011a; Yuan et al., 2013). The balance between the two isoforms may be implicated in the genesis of aberrant signaling during pathology. For instance, intermittent hypoxia in rodents is associated with increased HIF-1α and reduced HIF-2α protein in the carotid body (Nanduri et al., 2009). In these conditions, carotid body chemoreceptor signaling to the adrenal medulla selectively upregulates HIF-1α expression, inducing catecholamine secretion and blood pressure rise (Peng et al., 2014; Kumar et al., 2015), the latter is eliminated by adrenal demedullation (Bao et al., 1997). Restoring the levels of HIF-2α also prevents oxidative stress and blood pressure increase during intermittent hypoxia exposure (Nanduri et al., 2009). This demonstrates the contribution of HIF-1α pathway in the carotid body and its influence in increasing blood pressure.

As angiotensin II stimulates the HIF-1α pathway (see for example, Imanishi et al., 2014; Luo et al., 2015) RAAS activation could potentially cause an imbalance between HIF-α isoforms in the carotid body. This is supported by the fact that carotid body sensitivity is reduced when angiotensin II AT_1_ receptors are blocked (see discussion above) (Li et al., 2007). Whether there is a direct link between HIF and erythropoiesis pathways with the cooperative oxygen sensing by the kidney and carotid body, is unknown and will be off great interest to be studied in further detail.

## Clinical perspective

Our hypothesis on the cooperative oxygen sensing by the kidney and carotid body in blood pressure control could be of great relevance in the pursuit of novel ways to treat hypertension and cardiovascular disease (see Box 1). Reducing or eliminating the activity of the carotid body specifically is emerging as a viable target in diseases in which there is autonomic imbalance such as hypertensive conditions. Potentially, in resistant hypertensive patients that do not respond to renal denervation, concomitant elimination of carotid body activity could have a therapeutic benefit, as proposed by McBryde et al. (2017). Currently, surgical removal of the carotid body is the only way to reduce carotid body activity chronically in humans. Targeting aberrant hypoxia-mediated activation of renal and carotid body afferent activity would be potentially highly effective clinically.

Hydrogen sulfide, H_2_S, a gaseous endogenous signaling molecule, is increasingly identified to be involved in numerous cardiovascular (patho)physiology (Snijder et al., 2014, 2015; Xie et al., 2016; Huang et al., 2017; Merz et al., 2017). In the kidney, H_2_S exerts significant diuretic, natriuretic and kaliuretic effects by raising glomerular filtration rate and inhibiting tubular sodium re-absorption (Xia et al., 2009). In the renal medulla, H_2_S acts as an oxygen sensor where its accumulation in hypoxic conditions helps to restore oxygen balance by increasing medullary blood flow, reducing energy requirements for Na^+^ transport, and directly inhibiting mitochondrial respiration (Beltowski, 2010). Interestingly both low H_2_S levels and mitochondrial dysfunction have been found in humans (Granata et al., 2009; Perna and Ingrosso, 2012) and in animal models (Aminzadeh and Vaziri, 2012; Perna and Ingrosso, 2012; Gong et al., 2016) with cardiovascular disease. However, it remains to be established if intervention aimed to improve H_2_S levels, e.g., AP39, which proved to specifically increase H_2_S in the mitochondria (Ahmad et al., 2016; Chatzianastasiou et al., 2016) can alleviate tissue hypoxia and reduce blood pressure.

Pre-clinical and clinical evidence suggests that Finerenone, a next-generation non-steroidal dihydropyridine-based aldosterone antagonist, may achieve equivalent organ-protective effects with fewer adverse effects and reduced levels of electrolyte disturbance (Kolkhof et al., 2014; Bramlage et al., 2016). The latter is important for its potential applicability for patient with impaired renal function. This in combination with the above mentioned unknowm relation of the mineralcorticoid receptor with cooperative sensing of hypoxia by the kidney and carotid body invites for further pre-clinical research of Finerenone for the treatment of cardiovascular and renal hypertensive disease.

The argument can be made that pharmacological intervention that mimics and enhances natural, physiological response to disease may be preferable to single protein regulation. A promising approach to protect organisms against hypoxia, is upregulation of HIFs, which results in a broad and coordinated downstream reaction, possibly increasing cellular tolerance to hypoxia and thereby alleviating the double windup of RAAS and sympathetic hyperactivity that is responsible to the hypertensive state. Indeed, pre-conditioning by HIFα protein stabilization conferred protection in several models of acute renal ischemia (Bernhardt et al., 2009; Jarmi and Agarwal, 2009; Yang et al., 2009; Wang et al., 2012; Koeners et al., 2014). Furthermore, HIF stabilizing compounds are currently being investigated in clinical trials as a treatment for anemia (Besarab et al., 2016; Holdstock et al., 2016; Pergola et al., 2016). However, a major concern for clinical use includes the “broad pharmacology” of HIF stabilization due to the upregulation of many genes, including proteins that have been targeted for inhibition by marketed drugs (e.g., vascular endothelial growth factor, cyclooxygenase−2), in all tissues some of which may not be hypoxic. A potential way to circumvent unwanted effects of systemic HIF stabilization is to develop novel hypoxia activated pro-drugs, which are currently under development for targeting hypoxia in cancer therapy (Wilson and Hay, 2011). Hypothetically these pro-drugs will only be activated in specifically targeted hypoxic tissues like kidney and/or carotid body and thereby being able to alleviate hypoxia-mediated renal and carotid body afferent signaling, unrestrained RAAS activation and hence reduce blood pressure in hypertension.

Theoretically, all these therapies are effective only in patients whose have prolonged and/or severe tissue hypoxia. We know that, for example in the kidney, tissue oxygenation can vary wildly within and between individuals and follows a diurnal pattern. The latter, possibly due to variations in oxygen delivery, which is known to be determined by renal blood flow and peaks in the active phase (Emans et al., 2017), can act as cue for circadian clock genes via the HIF pathway (Adamovich et al., 2017). Thus, it is important to identify patients with tissue hypoxia, i.e., more responsive to hypoxia-oriented therapies. We believe that Magnetic Resonance Imaging (MRI) like blood oxygenation-level dependent (BOLD) MRI (Pruijm et al., 2016) and hyperpolarized MRI (Laustsen, 2016; Laustsen et al., 2016) represent very exciting tools to help us to elucidate the role of tissue oxygen metabolism in hypertension and other cardiovascular diseases.

## Author contributions

DP and MK drafted manuscript; DP, WP, JP, and MK edited and revised manuscript, approved final version of manuscript, and ensured integrity.

### Conflict of interest statement

The authors declare that the research was conducted in the absence of any commercial or financial relationships that could be construed as a potential conflict of interest.
